# Targeting Chemoresistant Tumors: Could TRIM Proteins-p53 Axis Be a Possible Answer?

**DOI:** 10.3390/ijms20071776

**Published:** 2019-04-10

**Authors:** Alessio Valletti, Flaviana Marzano, Graziano Pesole, Elisabetta Sbisà, Apollonia Tullo

**Affiliations:** 1 Department of Basic Medical Sciences, Neuroscience and Sense Organs, University of Bari “Aldo Moro”-Policlinico, Piazza G. Cesare, 11, 70124 Bari, Italy; alessio.valletti@uniba.it; 2 Institute of Biomembranes, Bioenergetics and Molecular Biotechnology, National Research Council-CNR, Via Amendola 122/O, 70126 Bari, Italy; f.marzano@ibiom.cnr.it (F.M.); g.pesole@ibiom.cnr.it (G.P.); 3 Department of Biosciences, Biotechnology and Biopharmaceutics, University of Bari “A. Moro”, Via Orabona 4, 70126 Bari, Italy; 4 Institute of Biomedical Technologies, National Research Council-CNR, Via Amendola 122/d, 70126 Bari, Italy; elisabetta.sbisa@ba.itb.cnr.it

**Keywords:** chemoresistance, TRIM proteins, p53

## Abstract

Chemosensitivity is a crucial feature for all tumours so that they can be successfully treated, but the huge heterogeneity of these diseases, to be intended both inter- and intra-tumour, makes it a hard-to-win battle. Indeed, this genotypic and phenotypic variety, together with the adaptability of tumours, results in a plethora of chemoresistance acquisition mechanisms strongly affecting the effectiveness of treatments at different levels. Tripartite motif (TRIM) proteins are shown to be involved in some of these mechanisms thanks to their E3-ubiquitin ligase activity, but also to other activities they can exert in several cellular pathways. Undoubtedly, the ability to regulate the stability and activity of the p53 tumour suppressor protein, shared by many of the TRIMs, represents the preeminent link between this protein family and chemoresistance. Indeed, they can modulate p53 degradation, localization and subset of transactivated target genes, shifting the cellular response towards a cytoprotective or cytotoxic reaction to whatever damage induced by therapy, sometimes in a cellular-dependent way. The involvement in other chemoresistance acquisition mechanisms, independent by p53, is known, affecting pivotal processes like PI3K/Akt/NF-κB signalling transduction or Wnt/beta catenin pathway, to name a few. Hence, the inhibition or the enhancement of TRIM proteins functionality could be worth investigating to better understand chemoresistance and as a strategy to increase effectiveness of anticancer therapies.

## 1. Introduction

Chemotherapeutic agents have become widely applied for the treatment of various types of malignancies. Nonetheless, the complexity of the disease leads to considerable heterogeneity between patients with regard to response, leading to the necessity to design suitable therapies according to tumour and patient characteristics.

Indeed, cancer cells sometimes fail to respond to a specific chemotherapeutic regimen because of the presence of inherently resistant cells that harbour pre-existing random mutations, which are then selected for. This is called “intrinsic resistance”, and is usually linked to processes involved in tumorigenesis itself [1]. Nonetheless, cancer cells can also acquire drug resistance throughout the treatment (i.e., “acquired resistance”), and this is possibly the most important factor that determines success or failure in cancer therapy [1]. This unwanted side outcome of drug treatment is extremely multifactorial and, albeit several mechanisms have been described in different tumours, research is still trying to understand if this is a selective process (like intrinsic resistance) or a non-genetic adaptive one (or probably a combination of both) [2,3]. Nowadays, heterogeneity of cancer cells is a matter a fact, while it is a recent acquisition that this heterogeneity is not exclusively genetic but also “phenotypic-only”, meaning that tumour cells within the same genetic background can shift between several states, corresponding to cancer stem-cell-like or resistant states [3,4]. This aspect is extremely crucial as non-genetic adaptation would imply that some cancer cells may transiently acquire a resistant phenotype in a non-genetic manner, i.e., by modifying their gene regulatory networks by chance or induced by a cytotoxic stress [2].

Hence, paradoxically, chemo- and radiotherapy would promote an environment where best fitting cells may develop resistance and become more aggressive. Accordingly, an efficient therapeutic approach should consider both the selective and adaptive nature of cancer plasticity not only to enforce better cytotoxic therapies, but also to make these therapies as effective as possible [5].

After introducing chemoresistance and some of the mechanisms involved in its acquisition in cancer, in this review we will discuss the role of E3 ubiquitin ligases tripartite motif (TRIM) family of proteins in positively or negatively sensitizing cancer cells. As this function is elicited in most cases by regulating p53 stability and activity, we will focus on the functional interaction between p53 and TRIM proteins, involving different mechanisms that can lead to an increased or decreased degradation of p53, or to a different subset of target genes that can be transactivated. Other p53-independent mechanisms and the therapeutic potential of TRIM proteins for cancer therapy will be also discussed in the last part of this review.

## 2. Chemoresistance Mechanisms

The development of chemosensitizers, compounds that can selectively enhance the cytotoxicity of chemotherapeutic agents without affecting the sensitivity of normal tissues, is highly demanded. They are meant to act restoring a drug sensitive state by stabilizing it, or by restoring/bypassing the pathways that are usually genetically altered or non-genetically dysregulated in drug resistance acquisition mechanisms. In both cases, it is worth knowing how cancer cells acquire treatment resistance as it can arise at different levels. 

**Figure 1:**
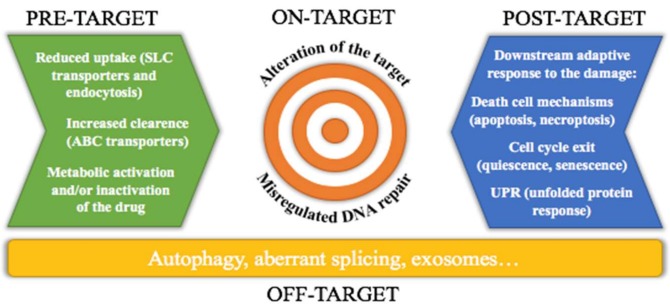
Scheme of the main chemoresistance acquisition mechanisms, grouped in pre-, on-, post- and off-target.

Indeed, a treatment can fail in its action simply because the active form of the drug can’t reach its intended target, or, even if it can, it can’t efficiently exert its action, e.g., induce a DNA damage. Nonetheless, resistance can be developed also because cells aren’t able to respond properly to the damage induced by the treatment, for instance as a consequence of either the malfunctioning of cell death pathways or the activation of pro-survival signalling pathways, even unrelated to the drug treatment per se, but which can abolish its death-inducing capacity [6,7]. Hence, most of the mechanisms of chemoresistance can be classified in pre-target, on-target, post-target and off-target (Figure 1).

Pre-target chemoresistance involves all those mechanisms affecting the amount of active drug inside the target cells (Figure 1). Indeed, the net intracellular concentration of a drug is a balance between its accumulation due to the uptake, and its clearance by the transporter-facilitated efflux or other mechanisms (e.g., exosomes) [8,9,10]. Another aspect to consider in pre-target chemoresistance is that some drugs require metabolic activation to manifest their anti-tumour activity, or that cancer cells can acquire or enhance their ability of inactivating the drug [11].

On-target chemoresistance involves all those mechanisms affecting the ability of the drug to interact with its target(s) or reducing the lesions it can directly determine (Figure 1). Hence, the main on-target resistance mechanism is the alteration of the specific target of a drug, by mutation or non-genetic misregulation of its expression, leading to the incapability of binding it or to a reduced effect of its action [12]. In a wider interpretation of target, the ability of cells to recognize and repair DNA lesions induced by some drugs can be included in the on-target mechanisms [13].

Post-target chemoresistance, involving either general or drug-specific mechanisms, acts by diminishing the efficiency of drug treatment by adaptive responses downstream of the drug target. Indeed, after the active drug has accumulated and acted on its target(s), the effectiveness of a treatment depends on how the cancer cell responds, typically by killing itself. Hence, most of post-target chemoresistance comes from the malfunctioning of cell death pathways, through the de-regulation of the extrinsic and intrinsic apoptotic pathways as well as their upstream regulatory networks, but also from the activation of alternative pro-survival signalling pathways (Figure 1) [12,13]. Indeed, cell signalling pathways are very complex and interlinked. Hence, when a targeted drug inhibits an oncogene, alternative pathways can reactivate and confer resistance to that particular drug, with tumour cells no longer depending on the original driving oncogene for uncontrolled cell division. Also UPR, namely Unfolded Protein Response, is an adaptive response cancers cells may operate when the exposition to a chemotherapeutic drug causes ER stress [14].

Finally, off-target chemoresistance involves all those mechanisms affecting molecular pathways not directly linked with the mechanism of a specific drug’s action or to the adaptive response of the cell (Figure 1). These mainly include autophagy, alternative splicing and exosomes exchanging resistant phenotype-inducing miRNAs and proteins between resistant and sensitive cells, eventually involving also tumour microenvironment cells [15,16,17,18].

The next three paragraphs will focus on the main chemoresistance acquisition mechanisms involving, directly or indirectly, members of the TRIM family.

### 2.1. Tolerance to DNA Damage and DNA Repair

The DNA represents the major intracellular target of many commonly used chemotherapeutic agents. For instance, cisplatin cytotoxicity is due to its binding to DNA leading to the formation of DNA inter- and intra-strand adducts [19]. Hence, on-target resistance to cisplatin is not linked to alteration of DNA but to impaired recognition of cisplatin-induced lesions, increasing tolerance to them or the ability to repair them [20]. These last two chemoresistance mechanisms, in particular, may be induced in different ways: (i) increasing directly the expression of DNA repair proteins (e.g., high expression of O^6^-methylguanine-DNA-methyltransferase (MGMT), involved in direct reversal repair, is associated with resistance to alkylating agents, in particular temozolomide [21]); (ii) over-expressing growth factor receptors, such as epidermal growth factor receptor (EGFR) and insulin-like growth factor 1 receptor (IGF1R), whose signalling pathways are involved in promoting different DNA repair mechanisms [22,23]. To chemosensitize cancers to DNA-damaging agents, several strategies are possible according to the DNA repair pathway affected. For instance, trans-communic acid, mahureone or masticadienonic acid are inhibitors of Polβ, a specialized DNA polymerase involved in translesion synthesis, and can sensitize to cisplatin those tumours characterized by the overexpression of this polymerase [24].

### 2.2. Apoptosis and Cell Cycle Regulation

Resistance to chemotherapy can arise when anti-apoptotic proteins, such as anti-apoptotic BCL-2 family members, inhibitors of apoptosis proteins (IAPs) and the caspase 8 inhibitor CASP8 and FADD like apoptosis regulator (CFLAR alias FLIP), undergo gain-of-function mutations or amplification or are overexpressed [25,26]. Moreover, these genes may also be transcriptional targets for pro-survival transcription factors, for example nuclear factor-κB (NF-κB) and signal transducer and activator of transcription 3 (STAT3), whose expression is frequently activated during tumorigenesis [25]. In humans, another important regulator of post-target resistance is p53 whose activation, when no mutations on its gene occur, can induce robust cell apoptosis, even in the presence of strong survival signals. Tumours harboring *TP53* mutations, which lead to expression of inactive p53 protein, account for about 50% of all human cancers. In another additional 40% of tumours, the p53 pathway is inactivated by alterations in its regulators [27]. All these cancers are associated with chemoresistance and, in general, predict a considerably worse patient prognosis. The role of p53 in chemoresistance will be discussed in more detail afterwards in connection with TRIM family proteins, as most of its members may regulate drug response in a p53-dependent manner.

A modification of the cell cycle may also be involved in the post-target resistance. Some chemotherapeutic drugs preferentially target cancer cells because of their higher cell division rate, hence a mechanism by which cancer cells may acquire resistance is driving the adaptive response following the action of the drug on its own target to a state of quiescence or senescence, slowing down or stopping the cell cycle [25]. Growth arrested cells may provide a source for relapse, as temporarily arrested cells (quiescent) can re-enter the cell cycle, and even permanently arrested cancer cells (senescent) can contribute to a pro-tumorigenic milieu by secreting factors that can stimulate the outgrowth, dissemination, and metastasis of drug resistant cancer cell clones or foster the survival of drug-sensitive cancer cells [28,29].

### 2.3. Autophagy

There is an increasing focus on the controversial role of autophagy in therapy resistance. While it has been identified as a cellular pro-survival process with a cell protective function, and autophagy inhibitors are reported to act synergistically with some anti-cancer treatments, autophagy is also the first step to apoptosis and initiators of autophagy are reported to restore drug sensitivity. Hence, autophagy can be functionally classified in cytoprotective, cytotoxic, and non-protective depending on whether its blockage leads to cell death, cell survival, or no effect respectively, and cytostatic when its induction results in cell growth arrest [15]. Obviously, the approach to reverse this kind of acquired resistance is defined by the specific autophagic mechanism acting in that cancer. For instance, cytoprotective autophagy can be inhibited by molecules like hydroxychloroquine that revealed its efficacy when administered in association with several targeted drugs [30,31], while cytotoxic autophagy can be induced by several drugs and natural compounds like vitamin D and curcumin [32].

## 3. TRIM Proteins as Chemosensitizers

As described so far, chemoresistance of cancer cells involves a large number of processes and interactions of multiple genes. One of the most pivotal regulatory processes relating to all proteins involved in chemoresistance pathways (pre-target, on-target, post-target and off-target) is the ubiquitin proteasome pathway. Indeed, ubiquitylation is one of the many post-translational modifications used by eukaryotic cells to regulate cellular physiology, and the ubiquitin-mediated proteolytic pathway has a crucial role in the elimination of regulatory proteins involved in cell cycle regulation, cellular signalling, DNA repair, apoptosis, morphogenesis, transport, protein quality control and transcriptional regulation. Emerging clinical evidence shows that the deregulation of ubiquitin-mediated degradation of oncogene products or tumour suppressors is likely to be involved in the etiology of cancers. Hence, the study of the involvement of E3 ubiquitin ligase activity in chemoresistance attracted increasing attention to a better understanding of the molecular basis and to provide novel therapeutic opportunities. 

TRIM proteins, that represent the largest class of RING-containing E3 ubiquitin-ligases, are increasingly emerging as crucial players in a variety of cellular functions, including cell growth, differentiation, immune response, carcinogenesis [33].

### 3.1. TRIM Family Proteins Structure and Function

TRIM proteins are defined by the presence of a tripartite motif (so-called RBCC motif) composed of a RING domain, one or two B-box motifs, a Coiled-Coil region [33,34]. The characteristic combination and order of these domains is highly conserved, suggesting that this minimal structure was selectively maintained to meet requirements of specialized functions and that the tripartite motif is an integrated functional structure, rather than a collection of separate modules [33]. The conservation of these domains in TRIM proteins from various species indicates that the RBCC motif is the key feature of the superfamily. TRIM proteins are conserved throughout the metazoan kingdom and have expanded rapidly during vertebrate evolution [35]. There are to date more than 80 known TRIM proteins in human and mice, classified into eleven subfamilies depending on differences in their domain structure [36]. There are few family members that do not have a RING domain and are still considered TRIM/RBCC because the rest of the motif (i.e., B-boxes and coiled-coil) is conserved in order and spacing. Functionally, the RING finger domain is involved in the ubiquitination system, mediating the transfer of ubiquitin from E2-Ub ligase enzyme to its substrates: this domain is therefore a characteristic signature of many E3 ubiquitin ligases [37]. It is noteworthy that the RING domain is not found in prokaryotes, consistent with the lack of the ubiquitination system in these organisms [33].

One of the peculiarities of the proteins belonging to this family is the variety of roles exerted by each of its members. The involvement of TRIM/RBCC proteins in such a lot of different functions, as well as in apoptosis, cell cycle regulation, senescence, differentiation, specific metabolic pathways, meiosis, can be attributed to the control of specific substrate levels through their ubiquitination activity [33]. This characteristic is also due to their structure that gives them the capability to fulfil both structural and functional tasks.

Through the interaction of Coiled Coil regions, the TRIM proteins associate in high molecular weight complexes that localize in proper sub-compartments identifying subcellular niches [38]. The great majority of TRIM proteins homo-interact, so each TRIM can define discrete cytoplasmic or nuclear structures: they can assume a cytoplasmic ribbon-like structure (TRIM29), create ‘cytoplasmic bodies’ of variable size (TRIM4, 5, 6, 9, 10, 12, 14, 21, 22, 23, 26, 27, 30 and 32), be located around the nucleus (TRIM13) or can localize to structures best described as ‘nuclear bodies’ (TRIM8, 19, 30 and 32) or ‘nuclear sticks’ (TRIM6). Members containing the bromo-domain such as TRIM24, 28 and 33 localize in the nucleus, associated with specific chromatin regions, as expected since the bromo-domain interacts with the acetylated lysines of histones [39].

It is possible that some TRIM/RBCC proteins may play a role in the “non-proteolytic” function of ubiquitin and ubiquitin-like modifications [40]. In fact, proteins tagged by ubiquitin molecules are not directed exclusively to the proteasome-mediated degradation, but they can be stabilized or re-localized in different cellular compartments by such modifications. The length of the ubiquitin chain, the lysine of ubiquitin involved in the bound, and the type of ubiquitin-like (UBL) molecule (SUMO, Nedd8, ISG15, etc.) covalently linked to the target define the downstream process [41]. Non-proteolytic ubiquitin-dependent modifications could also influence transcriptional activity when these modifications affect histone proteins, specific domains of some transcription factors, and the recruitment of proteasome subunits right to promoter regions [42]. On the other side, the TRIM/RBCC capacity to create higher order structures might serve as scaffold-maker, organizing an “ubiquitination system” in which E3s, deubiquitination enzymes, ubiquitin recognizing proteins and substrates can co-localize to simplify their interaction and to rapidly react to cellular stimuli [43].

### 3.2. TRIM Proteins in Cancerogenesis and Chemoresistance

Taking into account all the functions previously described, it is not surprising that mutations in *TRIM* genes or alterations in their protein functions are involved in several human diseases, first of all cancers. Indeed, TRIM proteins are implicated in various aspects of tumorigenesis, including proliferation, apoptosis, autophagy, transcriptional regulation, chromatin remodeling, invasion, metastasis and chemoresistance [44,45]. This feature is often associated with translocation of *TRIM* genes and creation of oncogenic fusion products, as in the case of TRIM19/PML, TRIM27/RFP and TRIM24/TIF1α, but they can influence cancer progression also per se [33].

As described before, cancer cells may acquire resistance to chemotherapy, or may have a high basal level of resistance, through a variety of mechanisms, among which the abrogation of apoptosis or cell cycle arrest due to mutation or inactivation of the tumour suppressor gene *p53* certainly represents a crucial point in the evolution of cancer towards chemoresistance. Indeed, p53 controls transcription of genes that are involved in cell cycle control, induction of cell death, senescence, cellular metabolism, autophagy, programmed cell death, DNA repair. Cells that lack functional p53 are unable to respond suitably to cellular stress, they accumulate mutations that favor the development of tumours and resistance to chemo- and radio-therapy. Hence, patients with a mutated or deregulated p53 network are more prone to not respond to chemotherapy, resulting in metastasis. 

The expression of some *TRIM* genes is promoted directly by p53 as *TRIML2*, *TRIM3*, *TRIM8*, *TRIM19*, *TRIM22*, *TRIM24* and *TRIM32*, and some of them, in turn, can regulate the activity and stability of p53. For *TRIM22*, in spite of being a p53 target gene, a controversial role in tumour progression as oncogene and tumour suppressor has been reported [46]. Moreover, an ever-increasing number of TRIM proteins function as negative or positive regulators for p53, with different effects of chemoresistance depending on the p53 signalling pathway affected (Table 1 and Table 2).

#### 3.2.1. p53 Positive Regulatory TRIM Proteins

**Figure 2:**
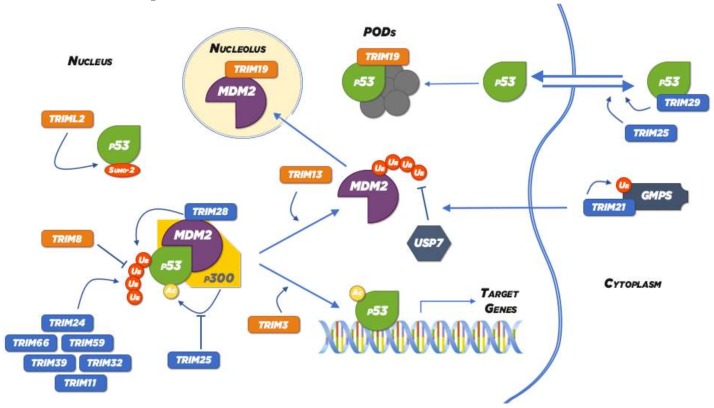
Schematic representation of the molecular mechanism by which TRIM family members can regulate p53 stability and activity. As indicated in the text, TRIM proteins can act as positive (in orange) or negative (in blue) regulators of p53. Arrows and T bars originating from TRIM proteins indicate if they stimulate or inhibit, respectively, a specific protein modification (e.g., ubiquitylation, which promotes p53 degradation or acetylation, which promotes p53 activation), the assembling/disassembling of a complex, or the re-localization of a protein.

Inhibition of the p53–degradation pathways, mainly by mouse double minute 2 homolog (MDM2) activity, is an important therapeutic strategy to stabilize p53 levels. The p53 suppressing activity of MDM2 can be targeted directly via two approaches: blocking the ubiquitin ligase activity of MDM2 and/or inhibiting the p53–MDM2 interaction. Some TRIM proteins, like TRIM19, TRIM13 and TRIM8 enhance the stabilization of p53 by interfering with MDM2 activity (Figure 2). 

TRIM19, which is encoded by the *promyelocytic leukemia* (*PML*) gene, is essential for the formation of subnuclear structures known as PML nuclear bodies (PML-NBs), also called promyelocytic oncogenic domains (PODs), which are thought to be sites of transcription, DNA repair and viral replication. Indeed, more than 100 proteins are sequestered in PML-NBs, including DNA repair-related proteins, transcription factors, and several enzymes. In particular, TRIM19 mediates the recruitment of p53 and modifying enzymes into these PML-NBs, which fosters p53 stabilization and post-translational modifications, such as CBP-dependent acetylation and Chk2-dependent phosphorylation that potentiate p53 function. It was also shown that TRIM19 enhances the stabilization of p53 by binding and sequestering MDM2 protein to the nucleolus. As TRIM19-deficient mice are resistant to the lethal effects of γ-irradiation and as DNA damage-induced apoptosis is prevented in TRIM19-negative cells, it is likely that TRIM19 is an obligatory component of p53 activation in response to DNA damage. Furthermore, TRIM19 is fused with retinoic acid receptor-α (RARα) in the t(15;17) translocation that specifically occurs in acute promyelocytic leukemia (APL). Intriguingly, this fusion protein induces deacetylation and degradation of p53 instead of protecting it from degradation [60].

TRIM13, also called Ret finger protein 2 or *LEU5* (*leukemia associated gene 5*), is an unstable protein involved in endoplasmic reticulum-associated protein degradation, that co-localizes with MDM2 in nuclear structures and mediates its polyubiquitination and proteasomal degradation in a RING-domain-dependent manner resulting in increased p53 stability and activity. It is worth noticing that TRIM13 is induced by γ-irradiation and that its overexpression induces apoptosis, while it is frequently deleted in B-cell chronic lymphatic leukemia and its downregulation decreases tumour cell growth in multiple myeloma [59,93,94].

TRIM8 mediates growth suppression only in a p53 wild-type background and this decreases in cell proliferation depends on the RING domain. Indeed, under stress conditions, p53 induces the expression of TRIM8, which in turn directly interacts with p53 inducing its stabilization by impairing the interaction with the negative p53 regulator, MDM2. Consequently, p53 induces the transcription of the genes involved in cell cycle arrest (e.g., *p21*, *GADD45*). Interestingly, TRIM8 down-regulation has been observed in a wide range of chemo- and radio-therapy resistant neoplasms, as in glioma, in anaplastic thyroid cancer (ATC) and in clear cell Renal Cell Carcinoma (ccRCC) [53,54,55]. Most importantly, it has been demonstrated that the recovery of TRIM8 expression in ccRCC-derived cell lines was able to induce a significant p53-dependent reduction in the proliferation rate, making cancer cells sensitive to different chemotherapy drugs as Nutlin-3, Cisplatin, Axitinib and Sorafenib [56]. TRIM8 down-regulation in ATC tissues is significantly correlated with the upregulation of miR-182 [54], while in ccRCC with the up-regulation of miR-17-5p and miR-106b-5p, belonging to the miR-17-92 family. Interestingly, a high expression level of MYCN transactivates the miR-17-92 and miR-106b/25 clusters, which in turn, down modulates other different targets, such as the tumour suppressor *p21* and *PTEN*, contributing to tumorigenesis. MYCN is a direct target of miR-34a, whose expression is activated by p53 [56]. Therefore, the down-regulation of the MYCN-miR-17-92 network in order to increase the TRIM8 expression level in cancer cells represents an interesting approach to inhibit uncontrolled cell proliferation and tumour growth and sensitize cancer cells to chemo- and radiotherapy in vivo.

Also TRIM3, that has been reported as a tumour suppressor in different cancers, such as glioblastoma, liver, cervical and colorectal cancers, exerts (at least partially) its role in controlling cell proliferation, migration and invasion of cancer cells by increasing p53 stability (Figure 2), with concomitant induction of transcriptional activity of downstream target genes, *p21* and *GADD45* [48]. It is worth noticing that TRIM3 can also directly interact with p21, sequestering it away from cyclin D1–cdk4 and consequently reducing proliferation [49]. Furthermore, it has been recently demonstrated that TRIM3 influences p38 MAPK signalling pathway, inactivating it, with negative effects on cell proliferation [50]. However, it is known that p38 phosphorylates and activates p53 when a DNA-damaging drug is used for chemotherapy, and the blockade of p38 leads to a decreased apoptotic response to anticancer agents. Indeed, the outcome of the inactivation of p38 signalling pathway, that can be mediated also by TRIM3, strongly depends on the cellular context, and specifically on the presence of a mutated or wild-type p53: in the first case, TRIM3 action contributes to chemoresistance to genotoxic drugs suppressing apoptosis, while in the latter it can suppress cell proliferation increasing the response to the chemotherapeutic agent [51,52].

As reported above, besides their ubiquitin-ligase activity, some TRIM proteins function as ligases also for SUMO-1 and SUMO-2/3. TRIML2, which is a p53 target preferentially induced by the p53-R72 variant, involved in the sumoylation of p53 (SUMO-2) (Figure 2), and this modification can shift the subset of genes transactivated by p53 towards proapoptotic target genes associated with prolonged oxidative stress, while reducing the transactivation of growth arrest genes [47]. High levels of TRIML2 has been reported in human oral cancer [95].

#### 3.2.2. p53 Negative Regulatory TRIM Proteins

On the other hand, other TRIM proteins, including TRIM11, TRIM21, TRIM24, TRIM25, TRIM28, TRIM29, TRIM31, TRIM32, TRIM39, TRIM59 and TRIM66, act as negative regulators of p53. 

Most of them act on MDM2 increasing its ubiquitin ligase activity, as TRIM21and TRIM28, or promote directly or indirectly the ubiquitination and degradation of p53 (Figure 2).

TRIM21, also known as Ro52, regulates p53 in a very elegant way involving the de-ubiquitylating enzyme USP7 and the guanine monophosphate synthase (GMPS). Normally, most GMPS is sequestered in the cytoplasm and its ubiquitination mediated by TRIM21 is crucial for its cytoplasmic retention. In this way, GMPS is kept separated from USP7 and p53 in the nucleus, where MDM2 binds and ubiquitylates p53, marking it for export from the nucleus and degradation by the proteasome. USP7 counteracts autoubiquitylation of MDM2, thereby promoting p53 degradation. In response to genotoxic stress or nucleotide deprivation, GMPS is released from the interaction with TRIM21, becomes nuclear and displaces MDM2 from the complex with p53 and USP7, which de-ubiquitylates p53 facilitating its stabilization [62,93]. TRIM21 low level are involved in hepatocellular carcinoma carcinogenesis [96].

TRIM28, also known as TIF1β interacts with MDM2 targeting p53 for proteasomal degradation. To date, several studies demonstrate significant upregulation of TRIM28 expression in different cancer tissues, which correlates with worse overall patient survival, suggesting that TRIM28 supports cancer progression. Indeed, TRIM28 has recently been correlated with increased epithelial mesenchymal transition (EMT), recurrence, metastasis and chemoresistance in non–small cell lung carcinoma, gastric, thyroid and in breast cancer [87]. In addition, MAGE proteins (Melanoma Antigen), which are upregulated in many cancers, were reported to function as cofactors in TRIM28-mediated p53 suppression. MAGE proteins bind to TRIM28 and induce the formation of a ternary complex composed of TRIM28, MDM2 and p53, leading to the suppression of p53-mediated apoptosis. However, opposite conclusions were also reported. In early-stage lung cancer, higher expression of *TRIM28* gene is associated with better overall survival, suggesting that TRIM28 may have also antiproliferative activity within tumour cells [70]. In fact, TRIM28 interacts with the pro-survival factor BCL2 related protein A1 (BCL2A1) mainly at the level of the mitochondria, promoting its degradation. BCL2A1 is an anti-apoptotic member of the BCL-2 family that contributes to chemoresistance in a subset of tumours. By inducing the ubiquitination and degradation of the pro-survival factor BCL2A1, TRIM28 may have an anti-tumoral activity in cancer cells whose survival depends on a high expression of BCL2A1. Interestingly, another TRIM protein, TRIM17, binds the TRIM28/BCL2A1 complex, disrupting it and inhibiting the ubiquitination and proteasomal degradation of BCL2A1. Therefore, overexpression of TRIM28 or downregulation of TRIM17 reduce the protein level of BCL2A1 and restore sensitivity to *B-Raf proto-oncogene*, *serine/threonine kinase* (*BRAF*)-targeted therapy in melanoma cells that exhibit a survival dependency on BCL2A1 [71].

Among TRIM proteins that directly target p53 for degradation, there are TRIM24, TRIM39, TRIM59, TRIM32 and TRIM31 (Figure 2).

TRIM24, also known as transcription intermediary factor 1α (TIF1α), regulates p53 by a mechanism similar to that of the master regulator of p53 stability, MDM2. Indeed, ATM kinase induced by DNA damage phosphorylates both p53 (Ser15) and TRIM24 (Ser768). On TRIM24 this modification leads to its autoubiquitination, like MDM2, and finally to its degradation, actually preventing its destabilizing function on p53. Like MDM2 again, *TRIM24* is also a transcriptional target of p53. Hence, when DNA damage has been fixed and p53 is no longer required, TRIM24 is not phosphorylated and can promote the degradation of p53 [65]. TRIM24 is highly expressed in gastric cancer conferring chemoresistance [86].

Similarly, also the E3-ubiquitin ligase *TRIM32* was identified as a p53 target gene whose inducible expression levels are regulated by p53 only in stress conditions. On the other side, TRIM32 is involved in the degradation of p53 through a negative feedback loop, impairing the p53 functions that promotes tumorigenesis [75].

TRIM39, previously identified as a regulator of the anaphase-promoting complex (APC/C), was recently shown to be able to bind and ubiquitylate p53. The relative importance of TRIM39 over MDM2 in regulating p53 stability seems to be cell-type dependent. Accordingly, for several cell lines that are relatively insensitive to nutlin-3a, an inhibitor of MDM2, depletion of TRIM39 increases apoptotic cell death [77]. Contrary to what was expected, TRIM39 can also stabilize the main growth-arresting target gene of p53, i.e., p21, by preventing its interaction with Cdt2 and blocking ubiquitylation and proteasomal degradation of p21 mediated by CRL4^Cdt2^ E3 ligase, as part of the APC/C pathway and in response to DNA damage [78]. 

TRIM59 recently has been implicated in the carcinogenesis of several cancers such as lung cancer, gastric, colorectal, prostate, bladder, breast and cervical cancers and promotes chemoresistance, through regulation of AKT and p53 pathways. Indeed, TRIM59 physically interacts with p53, promoting its ubiquitination and degradation [79]. Its overexpression has been found significantly associated with advanced TNM stage and lymph node metastasis and with absence of estrogen and progesterone receptors in breast cancers. Importantly, the level of TRIM59 overexpression in triple negative breast carcinoma (TNBC) is higher than that in non-triple negative breast carcinoma. These correlations suggest that tumours with TRIM59 overexpression are less likely to respond to endocrine therapy. TRIM59 reduces also the activation of caspase family proteins through inhibiting of mitochondria dependent pathway, while further investigation indicates that TRIM59 could upregulate Bcl-2 and Bcl-xL, with increase of AKT phosphorylation [80].

TRIM31 promotes anoikis-resistance (a form of programmed cell death that occurs in anchorage-dependent cells when they detach from the surrounding extracellular matrix) by targeting p53 for degradation. Indeed, TRIM31 directly promotes K48-linked poly-ubiquitination and proteasomal degradation of p53, subsequently overactivating AMPK pathway. In the metastatic process, anoikis-resistance is the prerequisite for cancer cells to survive in the circulating system and form a distant metastatic lesion [73].

Two TRIM proteins, despite lacking the RING-domain, are still able to negatively regulate p53, i.e., TRIM29 and TRIM66 (Figure 2).

TRIM29, also known as Ataxia-telangiectasia group D, was identified for its capacity to induce resistance to ionizing radiation (IR) in cells derived from patients with ataxia telangiectasia, a disorder characterized by ATM (Ataxia Telangiectasia-Mutated) deficiency. TRIM29 lacks a RING domain and has no E3 ubiquitin ligase activity, nonetheless it associates with p53 and sequestrates it in the cytoplasm thus inhibiting p53-dependent transcriptional activation. Moreover, TRIM29 prevents p53 acetylation mediated by histone acetyltransferase Tip60 (Tat-interactive protein 60), mediating its degradation. Interestingly, TRIM29 is highly expressed in multiple tumour types and is typically a marker of invasive/aggressive tumours, including lung, bladder, gastric, colorectal, ovarian, and endometrial cancers and in multiple myeloma with poor histological grade, large tumour size, great extent of tumour invasion and lymph node metastasis [72]. It is worth noticing that TRIM29 binds also the DNA repair factor RNF8 (Ring finger protein 8), which is required for robust double strand break DNA repair, and thus may be a determinant of resistance to both cytotoxic chemotherapy and ionizing radiation [88].

A further RING-less TRIM protein that controls p53 abundance is TRIM66, also known as transcription intermediate factor 1 delta. Overexpression of TRIM66 was found in hepatocarcinoma, in osteosarcoma and in non-small cell lung cancer, where it is associated with metastasis, chemoresistance and poor survival [81,90,91]. Its downregulation increases the abundance of p53 and the apoptosis markers caspase 7 and caspase 9, but the mechanism by which this regulation is exerted is still unknown, albeit TRIM66 bromo-domain being possibly involved [81].

Finally, other TRIM proteins influence the stability and activity of p53 through pathways that do not directly involve their ubiquitin ligase activity, as TRIM11 and TRIM25 (Figure 2).

TRIM11 was initially found to be important in nervous system function and in IFNβ production and antiviral activity, restricting HIV-1 replication and autophagy. Later, overexpression of TRIM11 was found in high-grade gliomas, lung cancer, liver and pancreatic cancer with an oncogenic function promoting cell growth, migration, invasion and chemoresistance, suggesting TRIM11 as a novel target for tumour treatment [85]. This oncogenic function is due to the action of TRIM11 in promoting p53 down-regulation with repercussions on p53 downstream pathways [61]. 

An interesting example is the estrogen-responsive TRIM25 that regulates p53 at different levels and with different mechanisms. One of them involves TRIM25 ability to interfere with the formation and activity of the ternary complex p53-MDM2-p300, blocking both the polyubiquitination and the acetylation of 53. This results in an increase of the p53 levels, that is not active though, as acetylation is required for the transactivation of growth-arresting and pro-apoptotic target genes. It is evident that this activity of TRIM25 can affect, mitigating it, the p53-dependent response in the presence of DNA damage also when induced by some cancer treatments [68]. Furthermore, TRIM25 can negatively regulate p53 activity also by interacting with G3BP2 (GTPase-activating protein-binding protein 2), responsible, together with RanBP2, an E3 ligase for sumoylation, for the SUMO conjugation of p53 and its androgen-mediated nuclear export. This relocalization of p53 is also enhanced by a decreased activity of MDM2 probably due to G3BP2, leading to the monoubiquitination (required for nuclear export) instead of polyubiquitination of p53 [69].

### 3.3. p53-Indipendent Resistance Acquisition Mechanisms Correlated to TRIM Proteins

Besides p53 regulation, TRIM proteins are implicated in a variety of cancer signalling pathways, acting as regulators of resistance acquisition or sensitization mechanisms in different cancers. In the case of TRIM14 and TRIM37, up until now there has not been any reported correlation with p53, and the mechanisms they are involved in are connected to chemoresistance.

TRIM14, one of the few TRIM family members lacking the RING domain, is markedly upregulated in oral tongue squamous cell carcinoma and correlated cell lines, where it induces epithelial-mesenchymal transition (EMT) and the formation of cancer-initiating cells (CICs) with a resistant phenotype. It has been seen that in this case chemoresistance can be reversed by the action of miR-15b targeting TRIM14 and inducing cisplatin-induced apoptosis [92]. Furthermore, the overexpression of TRIM14 also explains the chemoresistance to temozolomide in gliomas. In this case, the underlying mechanism requires the post-transcriptional stabilization of Dvl2 mediated by its direct binding to TRIM14 C-terminal domain. This interaction induces the activation of the canonical Wnt/β-catenin pathway and, in particular, the expression of MGMT (O^6^-methylguanine DNA methyltransferase) involved in the mechanism of direct reversal repair of DNA damage induced by alkylating agents such as temozolomide [82].

TRIM37, if overexpressed, confers resistance to the DNA-damaging anticancer drug cisplatin in vitro and in vivo through the activation of the NF-κB pathway. In detail, genotoxic stress-activated ATM kinase directly interacts with and phosphorylates TRIM37 in the cytoplasm, which induces translocation of TRIM37 into the nucleus. Here, TRIM37 forms a complex with NEMO (NF-kB essential modulator) and TRAF6 (TNF receptor associated factor 6) via a TRAF6-binding motif (TBM). In this complex, TRIM37 monoubiquitines NEMO at K306, consequently resulting in nuclear export of NEMO and IKK/NF-κB activation. A strategy to target and block this ATM/TRIM37/NEMO axis has been developed via a cell-penetrating HIV-1 transactivator of transcription (TAT)-conjugated TRIM37/TBM (TAT-TRIM37/TBM) peptide which can compete for the formation of the complex including TRIM37, resulting in a hypersensitivity of cancer cells to genotoxic drugs [83].

For other TRIM proteins, p53-independent mechanisms act synergically or alternatively to those somehow correlated to p53 seen in the previous paragraphs. Indeed, most of them are regulators of master pathway involved in tumorigenesis and chemoresistance, e.g., NF-κB, STAT and Akt pathways.

For instance, TRIM8 is part and parcel of at least two pathways whose role in inducing chemoresistance is well-known, i.e., NF-κB and STAT3 pathways [97,98]. First, TRIM8 mediates the activation of NF-κB by directing PIAS3 (Protein Inhibitor of Activated STAT-3) to degradation after ubiquitination, thus preventing its interaction with p65, one of the five components that form the NF-κB [57]. Furthermore, TRIM8, in response to TNF, activates TAK1 (TGF-β activated kinase-1) by K63-linked polyubiquitination, determining IKK-mediated activation of the NF-κB pathway [58]. Second, the degradation of PIAS3 mediated by TRIM8 also affects STAT3 [57], besides its capability to decrease the protein stability of SOCS-1 (suppressor of cytokine signalling-1), thus preventing it from inhibiting the JAK-STAT activation induced by interferon-γ [99].

Both TRIM31 and TRIM32 can confer chemoresistance through the activation of NF-kB signalling. In particular, TRIM31 up-regulates the levels of nuclear p65 by promoting K63-linked polyubiquitination of tumour necrosis factor receptor-associated factor 2 (TRAF2) and sustains the activation of NF-κB in pancreatic cancer cells. Indeed, high TRIM31 expression is associated with an aggressive phenotype and poor prognosis in pancreatic cancer patients. Moreover, TRIM31 overexpression confers gemcitabine resistance on pancreatic cancer cells [74]. Similarly, TRIM32 induces cisplatin resistance in breast cancer and non-small-cell-lung cancer, through the regulation of mitochondrial function and NF-κB/Bcl-2 signalling pathway, probably upregulating IkB phosphorylation status [76,89]. For this reason, the overexpression of TRIM32 is correlated with poor prognosis.

TRIM24, thanks to its bromodomain, can bind the promoter of catalytic subunit alpha of the phosphatidylinositol-4,5-bisphosphate 3-kinase (PIK3CA) to activate its expression, enhancing PI3K/Akt signalling. This activation also affects NF-κB and results in the regulation of the expression of the DNA repair enzyme MGMT, determining temozolomide resistance [66]. TRIM24 can also sense the non-canonical H3K23ac histone modification specifically upregulated by EGFR activation, and acts as transcriptional co-activator recruiting STAT3 to chromatin [67].

TRIM21 can regulate response to chemotherapeutic treatment exploiting less conventional pathways. For instance, in pancreatic cancer and colon cancer cell lines, TRIM21, in response to cisplatin, downregulates *Par-4* (*Prostate apoptosis response protein 4*), a tumour suppressor gene inducing cancer cell specific apoptosis, hence increasing the resistance to this drug [63]. Furthermore, TRIM21 can ubiquitinylate acetylated fatty acid synthase (FASN) promoting its degradation [64]. Albeit the detailed molecular mechanism by which FASN induces chemoresistance is still unclear, it is known that its increased expression can upregulate Pyruvate Kinase M2 (PKM2) and enhance Warburg Effect (inducing chemoresistance to gemcitabine and also radioresistance), and also protect from drug induced apoptosis by decreasing ceramide levels and thereby the activation of caspase 8 [100,101].

## 4. TRIM Proteins as Promising Targets for Overcoming Chemotherapy Resistance

As we have seen in this review, the heterogenous family of TRIM proteins plays crucial roles in the physiological homeostasis of the cell and in pathological processes. In particular, in many cancers the aberrant expression of TRIM proteins has been found, which fine-tunes the growth arrest versus cell death decision. It is noteworthy that several TRIM proteins have been found to regulate the stability and the transcriptional activity of p53 and as such are linked to cancer progression and chemoresistance. Moreover, since they act mostly as E3-ligases in the ubiquitin-proteasome system, they are ideal candidates for cancer therapy. The therapeutic inhibition of proteasome activity started in 2003 by using the small pharmacological agent bortezomib for the treatment of multiple myeloma [41]. Nonetheless, Bortezomib produces side effects, such as neuropathy, but the new-generation of proteasome inhibitors such as carfilzomib, NPI-0052 and MLN-9708 have improved pharmacological activity and reduced side effects [102]. Ideally drugs targeting individual E3 ubiquitin ligases should provide high levels of sensitivity with minimal side effects. For example, as we have seen above the rescue of TRIM8 protein levels in the aggressive and chemo-resistant clear cell Renal Cell Carcinoma by anti-miR-17, makes these cells sensitive to chemotherapy drugs like cisplatin, nutlin, sorafenib and axitinib [56]. The use of miRNA-based therapeutics (miRNA antagonists and miRNA mimics) is now widely recognised. In the last 10 years, slightly more than 1000 human unique miRNAs have been discovered and await utility in clinical applications [103]. In particular, some of them have already been validated in clinically-relevant animal models, a few are in (pre)clinical development, others need further extensive investigations for their medical use. Although, to date an effective miRNA delivery system is not yet available, the use of liposomal and polymer-based delivery technologies is encouraging and is expected to jump-start the clinical development of therapeutic miRNAs.

Moreover, to surmount the failure rate in chemotherapy and minimize its side effects, a re-emergence of studies on natural product for drug discovery is evident. Since the isolation of vinca alkaloids in the 1950s, a series of anticancer drugs, currently used in chemotherapy, are natural products derived from plants or marine organisms [104]. For example, MDM2-specific inhibitors, such as sumpervirine, isolissoclinotoxin B, diplamine B and lissoclinidine B, which inhibit E3 activity of MDM2 and increase the expression level of p53 are bioactive natural alkaloids isolated from the ascidian *Lissoclinum cf. badium* [105,106,107]. 

Nevertheless, post-translational modifications like ubiquitination, sumoylation and neddylation mediated by TRIM proteins can affect not only the stability but also the activity of target proteins. In particular, the functional consequence of ubiquitin-like modifications is just beginning to emerge, and ubiquitin-like modifications may provide new therapeutic targets in cancer in the future. For instance, neddylation pathways may be correlated with tumorigenesis as, for instance, it has been reported that TRIM40 is a putative tumour suppressor in gastrointestinal carcinomas, where is responsible for the neddylation of IKKγ and consequently for the inhibition of NF-κB-mediated cell growth [84]. 

In addition, enhancing the efficiency of conventional chemotherapy (chemosensitization) and preventing the developing of cancer (chemoprevention) remain another main goal of cancer research. Therefore, there is a great deal of rigorous effort in the identification of natural drugs and phytochemicals acting as chemosensitizers or chemopreventives, and in unravelling their molecular mechanism. An increasing number of clinical trials intended to evaluate the chemosensitizing efficiency of natural compounds are complete and/or ongoing (https://clinicaltrials.gov/ct2/home). Curcumin, genistein, quercetin, epigallocatechin gallate (EGCG), emodin, and resveratrol are the most reported phenolics which inhibit antiapoptotic pathways and prosurvival signals, thereby enhancing apoptosis, frequently by p53-dependent pathway, in various cancers [108]. In particular, curcumin, a polyphenolic phytochemical derived from the rhizomes of the Curcuma longa, is one of the best-studied plant derivatives in the world. Curcumin has been used as a therapeutic agent in the treatment of various types of disease due to its apoptosis-inductive, chemopreventive, anti-angiogenic and anti-invasive/metastatic properties as well as its low toxicity. Therapeutic strategies combining the use of curcumin with more conventional chemotherapeutic drugs enables the overcoming of drug resistance, improves clinical effects and decreases nephrotoxicity [109]. For instance, the combination of curcumin and IR treatment has been reported to increase radio- sensitivity of RCC cells by suppressing NF-kB signalling pathway, specifically by inducing apoptosis and G2/M cell cycle arrest, and inhibiting IR-induced DNA damage repair [110]. In the same cells, curcumin can also enhance their chemosensitivity to Temsirolimus, an mTOR inhibitor used as first-line treatment of metastatic RCC, by upregulating the YAP/p53 pathway [111]. The correlation between TRIM family and natural compounds like curcumin has not been studied yet, but some independent reports give hints that these compounds have some overlapping mechanisms with some TRIM proteins. For instance, as we have seen, TRIM8 induces the downregulation of PIAS3 activating STAT3 signalling pathway, while curcumin upregulates this inhibitor of STAT3 [57,112].

Covering the different mechanisms of action of TRIM proteins, it appears clear that their function is not limited to their E3-ligase activity, but it embraces a plethora of functions that are relevant in other chemoresistance acquisition mechanisms. For instance, recent reports indicate that TRIM proteins are involved in epigenetic regulation, as we have seen for example for TRIM24, suggesting that TRIM proteins could contribute to tumour suppression or development also by indirectly regulating gene expression [67]. In particular, targeting TRIM24 seems to be a potentially feasible strategy as recently at least two dual TRIM24/BRPF1 inhibitors derived from 1,3-benzimidazolone, acting on the sub-family V bromodomains, have been developed [113]. As we have discussed in the first part of this review, part of tumours chemoresistance can be explained by transitory alterations of some gene expression networks that somehow confer a proliferative advantage in the stress conditions induced by anticancer treatments. Some TRIM proteins like TRIM8, TRIM14, TRIM24, TRIM31, TRIM32, TRIM37 and TRIM40 can regulate the master pro-survival regulatory pathways of NF-κB and STAT3. This opens up new ways to inhibit these pathways, preventing cancer cells to exploit them to elude the cytotoxic effects of drugs affecting other pathways. For instance, a recent study demonstrated that a chimeric antibody targeting TRIM14 (called Chanti-TRIM) can successfully inhibit cell growth migration and invasion of osteosarcoma cells by inhibiting the MMP-induced NF-κB signalling pathway and chemosensitize osteosarcoma cells to cisplatin treatment by promoting apoptosis possibly by regulating the NF-κB signal pathway [114].

## 5. Conclusions and Future Perspectives

In conclusion, these multiple roles of TRIM proteins undoubtedly represent a good reason to better investigate TRIMs targeting as an integrative approach in cancer. Moreover, the use of natural compounds, combined with targeted drugs, could represent a new promising therapeutic protocol to improve clinical effects and decrease toxicity in various cancers.

## Figures and Tables

**Table 1:** TRIM proteins described in the manuscript are classified based on their capability to regulate positively or negatively p53, or to act by other p53-independent mechanisms. The * indicates which TRIM protein is also a transcriptional target of p53.

TRIM Proteins and Chemoresistance Pathways		Ref.
**p53 Positive Regulators**		
TRIML2 *	p53 sumoylation	[47]
TRIM3 *	p53 stabilization; p21 sequestration (preventing cyclin D1-cdk4 accumulation); p38 signalling pathway inactivation	[48,49,50,51,52]
TRIM8 *	Impairment of the interaction between p53 and MDM2; PIAS3 ubiquitylation (activation of NF-κB and STAT3 pathways); TAK1 activation (enhancement of the NF-κB pathway)	[53,54,55,56,57,58]
TRIM13	MDM2 polyubiquitylation and proteasomal degradation	[59]
TRIM19 *	Recruits p53 into the PML-NBs; Sequestrates MDM2 into the nucleolus	[60]
**p53 Negative Regulators**		
TRIM11	p53 down-regulation	[61]
TRIM21	GMPS ubiquitylation and sequestration into the cytoplasm; PAR-4 down-regulates PAR-4; FASN ubiquitylation for degradation	[62,63,64]
TRIM24 *	p53 ubiquitylation for degradation; Induction of the expression of PI3KCA (activation of PI3K/Akt and NF-κB pathways); Co-transcriptional activator (recruitment of STAT3)	[65,66,67]
TRIM25	Interferes with the formation of the complex p53-MDM2-p300; Relocalization of p53 into the cytoplasm by interacting with G3BP2	[68,69]
TRIM28	Interaction with MDM2 for targeting p53 for proteasomal degradation; Interaction (inhibited by TRIM17) with the anti-apoptotic BCL2A1 to induce its ubiquitylation and degradation	[70,71]
TRIM29	Sequestration of p53 into the cytoplasm; Degradation of Tip60 (inhibition of p53 acetylation); Binding to the DNA repair factor RNF8	[72]
TRIM31	K48-linked polyubiquitylation and proteasomal degradation of p53; polyubiquitylation of TRAF2 upregulating the levels of nuclear p65 (NF-κB)	[73,74]
TRIM32 *	Degradation of p53; Upregulation of the phosphorylation of IkB	[75,76]
TRIM39	p53 ubiquitylation for degradation; p21 stabilization (by preventing its interaction with Cdt2)	[77,78]
TRIM59	Enhancement of p53 ubiquitylation and proteasomal degradation; Reduction of caspases activation; Upregulation of Bcl-2 and Bcl-xL, increasing Akt phosphorylation	[79,80]
TRIM66	Down-regulation of p53 and caspases 7 and 9	[81]
**Other Mechanisms**		
TRIM14	Dvl2 binding and stabilization (activation of Wnt-beta catenin pathway and the expression of MGMT)	[82]
TRIM37	Nuclear export of NEMO (IKK/NF-κB activation)	[83]
TRIM40	Neddylation of IKKγ (inhibition of NF-κB-mediated cell growth)	[84]

**Table 2:** Tripartite motif (TRIM) proteins described in the manuscript are listed based on their role in chemoresistance to specific drugs, in different types of cancer. The arrows indicate if that TRIM protein was found up- or downregulated. ccRCC: clear cell Renal Cell Carcinoma; TS: Tumour Sample; XE: Xenograph; HCT116: Colon carcinoma cell line, HEK293T: human embryonic kidneys that expresses a mutant version of the SV40 large T antigen; GC: Gastric Cancer; BC: Breast Cancer; MDA-MB-231: epithelial human breast cancer cell line; BT-474: human breast ductal; carcinoma HEK293: human embryonic kidney; Panc1: human pancreatic cancer cell line isolated from a pancreatic carcinoma of ductal cell origin; BxPc3: human pancreatic cancer cell line; HPDECs: primary cultures of normal human pancreatic duct epithelial cells; MCF-7: human breast adenocarcinoma cell line; T-47D: human breast ductal carcinoma derived from metastatic site; HBE: human bronchial epithelial; A549: adenocarcinomic human alveolar basal epithelial cells; H1299: human non-small cell lung carcinoma cell line; H460: human large cell lung cancer (lung: pleural effusion); H358: lung/bronchiole, derived from metastatic site: alveolus; H3255, H1975, H2228: human lung adenocarcinoma; CAPAN2: human pancreatic ductal adenocarcinoma cell line; PC: Pancreatic Cancer; NSCLC: non-small-cell lung carcinoma; hTERT-RPE: epithelial cells immortalized with hTERT; SK-BR-3: human breast cancer cell line isolated by the Memorial Sloan–Kettering Cancer Center; Hep-3B, SNU-449: human liver hepatocellular carcinoma; HL-7702: human normal liver cell line; OTSC: oral tongue squamous carcinoma; SCC25: human tongue squamous carcinoma cell line; EC: esophageal cancer; PCL: primary cultures of normal esophageal epithelial cells; Eca109: human esophageal carcinoma epithelial cell.

TRIM-Mediated Chemoresistance in Cancers				
TRIM Proteins	Expression	Cancer	Chemotherapeutic Drug	References
TRIM8	↓	ccRCC-TS and XE	nutlin-3, cisplatin, axitinib and sorafenib	[55,56]
TRIM11	↑	HCT116 and HEK293T	proteasome inhibitor bortezomib (BTZ)16, autophagy inhibitor chloroquine (CQ)17, piperlongumine (PL) and celastrol	[85]
TRIM24	↑	GC-TS	5-fluorouracil	[86]
TRIM28	↑	BC-TS, MDA-MB-231 and BT-474	doxorubicin, 5-fluorouracil, and methotrexate	[87]
TRIM29	↑	HEK293, Panc1, BxPc3 and CAPAN2	cytotoxic chemotherapy and ionizing radiation	[88]
TRIM31	↑	PC-TS and HPDECs	gemcitabine	[74]
TRIM32	↑	BC-TS, MCF-7, T-47D / NSCLC-TS, HBE, A549, H1299, H460, H358, H3255, H1975, H2228	cisplatin	[76,89]
TRIM39	↑	hTERT-RPE	nutlin-3a	[77]
TRIM59	↑	BC-TS, MCF-7 and SK-BR-3	paclitaxel	[80]
TRIM66	↑	NSCLC-TS, Hep-3B, SNU-449 and HL-7702	cisplatin	[90,91]
TRIM14	↑	OTSC-TS and SCC25	cisplatin	[92]
TRIM37	↑	EC-TS and PCL, Eca109	cisplatin	[83]

## Abbreviations

TRIM	TRIpartite Motif
SLC	SoLute Carrier
MDR	MultiDrug Resistance
ABC	ATP-Binding Cassette
MGMT	O^6^-MethylGuanine-DNA-MethylTransferase
IAP	Inhibitors of Apoptosis Protein
CFLAR	CASP8 and FADD like apoptosis regulator
EGFR	Epidermal Growth Factor Receptor
IGF1R	Insulin-like Growth Factor 1 Receptor
STAT3	Signal Transducer and Activator of Transcription 3
NF-κB	Nuclear Factor κB
UPR	Unfolded Protein Response
PML	ProMyelocytic Leukemia
POD	Promyelocytic Oncogenic Domain
RARα	Retinoic Acid Receptor α
APL	Acute Promyelocytic Leukemia
ATC	Anaplastic Thyroid Cancer
ccRCC	Clear Cell Renal Cell Carcinoma
GMPS	Guanine MonoPhosphate Synthase
EMT	Epithelial Mesenchymal Transition
TIF1α	Transcription Intermediary Factor 1α
APC/C	Anaphase Promoting Complex or Cyclosome
IR	Ionizing Radiation
ATM	Ataxia Telangiectasia-Mutated
Tip60	Tat-Interactive Protein 60
CIC	Cancer-Initiating Cell
NEMO	NF-κB Essential Modulator
TRAF	Tumor necrosis factor Receptor Associated Factor
TAT	HIV-1 TransActivator of Transcription
TBM	TRAF6 Binding Motif
PIAS3	Protein Inhibitor of Activated STAT3
TAK1	Transforming growth factor β Activated Kinase 1
SOCS1	Suppressor of Cytokine Signalling 1
PIK3CA	PhosphatidylInositol-4,5-bisphosphate 3-Kinase subunit alpha
FASN	Fatty Acid Synthase
PAR-4	Prostate apoptosis response protein 4
EGCG	EpiGalloCatechin Gallate
